# A novel approach to wind energy modeling in the context of climate change at Zaafrana region in Egypt

**DOI:** 10.1038/s41598-025-90583-2

**Published:** 2025-03-04

**Authors:** Bassem Khaled Kamel, Almoataz Y. Abdelaziz, Mahmoud A. Attia, Amr Khaled Khamees

**Affiliations:** 1 Department of Electrical Power and Machines, Faculty of Engineering, Ain Shams University, Cairo, Egypt; 2 Faculty of Engineering and Technology, Future University in Egypt, Cairo, 11835 Egypt; 3 Department Engineering Physics and Mathematics, Faculty of Engineering, Ain Shams University, Cairo, Egypt

**Keywords:** Wind energy, Wind turbines, Temperature de-rating, Wind speed modeling, Probability distribution function, Exponential distribution optimizer (EDO), Electrical and electronic engineering, Energy infrastructure

## Abstract

Global warming, driven by the excessive emission of greenhouse gases from the combustion of fossil fuels, has emerged as a critical environmental challenge which is considered as a motivation for this research. Where, the switch to sustainable energy sources is crucial because of the pressing need to slow down climate change and lower carbon footprints. Of all the renewable energy sources, wind energy is particularly important as a means of reducing carbon emissions from the generation of electricity. With the increase in the penetration of renewable energy resources in electrical power systems, the stochastic behavior of the renewable energy resources has to be taken into account for better analysis in power systems. However, the stochastic behavior of the renewable energy is also affected by the environmental conditions. In this context, The main objective of this paper is to present a novel wind energy modeling that includes the effect of ambient temperature on the wind turbine capabilities. This effect is presented as the de-rating curve for wind turbine output power to respect the thermal capabilities of the electrical components of the wind turbine. That’s why this novel model is developed to consider the effect of ambient temperature to represent the practical limitations of wind turbines which wasn’t considered by previous literature although the temperature has a siginicant impact on the wind turbine output power. In this Paper, Gamesa G80 wind turbine is used to perform the numerical analysis of the proposed new model. Moreover, Exponential Distribution Optimizer (EDO), Aquila Optimizer (AO), and Equilibrium Optimizer (EO) algorithms are used to find various probability distribution functions (PDFs) parameters to model wind speed data from Zaafrana region in Egypt using Root Mean Square Error (RMSE) and Coefficient of Correlation (R^2) as judging criteria. In addition, real temperature data from the same site are used to validate the proposed model compared to the manufacturer’s capabilities. The results show that mixed PDFs provide a better representation for the wind speed data. Moreover, the study demonstrates that ambient temperature cannot be neglected in wind power modeling, as the wind turbine output power varies significantly. Additionally, this work highlights the impact of climate change on the efficiency of renewable energy sources like the wind energy. The proposed wind energy model could be valuable to system operators as a decision-making aid when dealing with and analyzing complex power systems.

## Introduction

Alerts as a result of global warming and growing environmental concerns have been raised significantly as a result of using fossil fuels to generate electricity. Furthermore, the development of renewable energy technology has been essential in establishing them as the most economical and ecologically viable options. Hence, the efficient control and operation of power systems might be significantly affected by the strategic integration of renewable energy resources in suitable areas this will imply potential adjustments to traditional power network characteristics^1–4^. One of the renewable energy resources with high penetration and noticeable share over the past decades is the wind energy conversion system where the wind energy is converted to mechanical energy then this energy is converted into electrical energy^5–7^. According to the International Renewable Energy Agency (IRENA), the installed capacity of wind energy conversion systems reached 1017.4 GW in 2023 compared to 563.68 GW in 2018^8^. With the continuous penetration of renewable energies, the stochastic behavior should be taken into account as it has a significant impact on electrical system stability, reserve scheduling, electrical losses and carbon emissions^9^. For better evaluation of wind energy in the electric networks, the stochastic characteristics for wind have to be taken into account where, this has to be done for every location with a potential for wind energy production through assessing the wind speed historical data through modeling the wind profile^10^.

Different PDFs have been used to model wind speed depending on each location such as Weibull, Lognormal, Gamma, Beta, Rayleigh and other distribution functions^11^. Many studies model renewable energy resources to predict the power generation for wind energy to maintain power system security considering wind power variability techniques, where several methods such as numerical, statistical^12,13^. Persistence, physical, statistical, and hybrid models were evaluated to accurately predict the available wind power Moreover, a new proposed model managed to provide a more accurate wind speed forecast^14,15^. Other techniques, such as kernel-density was also used to estimate the wind speed PDF in^16^.

The up-crossing rate method was used to estimate Rayleigh and Weibull PDFs parameters based on cumulative distribution function mapping, Rayleigh function was better compared to Weibull^17^. Numerical methods were used to estimate the Weibull and Gamma PDFs parameters for wind energy for modeling wind speed^18,19^. Maximum likelihood, modified maximum likelihood and Multi-objective moments methods were used to evaluate PDFs to model wind speed, the analysis showed that extended generalized Lindley and generalized gamma distributions had better representation^20,21^. The Power density (PD) method was also used as a numerical technique in^22^.

The modified maximum likelihood method was used to find the parameters for Weibull and Modified Weibull Distribution, the validation tests were performed using Chi-square and Kolmogorov-Smirnov, the results showed the superiority of Modified Weibull Distribution^23^. Moreover, Empirical, Moment and Energy pattern factor methods were used in^24^ to determine the parameters for Rayleigh and Weibull PDFs. Monte Carlo simulation (MCS) with its variations were used to find parameters for wind speed PDFs^18^. Quasi-MCS using truncated regular vine copula was more superior compared to numerical methods^25^. Metropolis–coupled Markov chain MCS was tested to predict the stochastic behavior of wind energy^26^. MCS combined with the K-means clustering method was proposed in^27^ and gave better results and reduced computational time.

Recently, Artificial Intelligence (AI) optimization algorithms are used over numerical methods to estimate PDFs parameters due to their higher accuracy. Genetic algorithm (GA), Bacterial Foraging Optimization algorithm (BFOA), Simulated Annealing (SA) were used to obtain Weibull PDF parameters showing better results over classical methods^28^. Firefly algorithm (FA) was used to obtain the Weibull PDF parameters in^29^. Whale Optimization algorithm (WOA) was used to obtain parameters for Weibull, Gamma and Rayleigh PDFs in^30^. Particle Swarm optimization (PSO), Cuckoo Search algorithm (CSA), Gray Wolf algorithm (GWA), Ant Colony optimization (ACO), Salp Swarm optimization (SSO)^31,32^, adaptive dynamic grey wolf-dipper throated optimization (ADGWDTO) algorithm^33^ were also used to obtain Weibull PDF parameters. Further approaches to better model wind speeds were implemented where, Two and three components mixture PDFs were evaluated based on Weibull, Gamma, and Inverse Gaussian PDFs. Aquila Optimizer (AO) and modern Mayfly algorithm (MA) were used to obtain he parameters for various PDFs combinations. The results showed that mixed PDFs managed to provide more accurate representation using root mean square error (RMSE), Chi-square error ($\:{X}^{2}$), and coefficient of Correlation ($\:{R}^{2}$) as judging criteria^34,35^.

Based on the previous studies, it can be found that there are some limitations on modeling the wind energy that can be summarized as:

- Lack of analysis of wind speed modeling using mixed PDFs.
- Lack of linkage between real wind turbine performance and theoretical models used in power system problem analysis.
- No consideration for the environment and ambient temperature impact on wind energy modeling.

To cover the previous gaps, this paper provides;

- Wind speed modeling using mixed PDFs combinations to represent wind speed accurately.
- Novel wind enery model to consider the effect of ambient temperature on wind turbine output power.
- A comparision between the classical and proposed wind energy models to show the contribution and achievements of the novel model.

In this paper, Weibull, Gamma, and Lognormal PDFs as well as their mixtures to accurately model the historical wind data in Zaafarana region in Egypt with a duration of three years. Where, Exponential Distribution Optimizer (EDO), Aquila Optimizer (AO), and Equilibrium Optimizer (EO) algorithms are used to find the optimum PDF parameters with the objective function of minimizing the root mean square error (RMSE). Moreover, the paper proposes a novel wind energy model incorporating the effect of the ambient temperature as the performance of wind turbine is reduced when wind turbine operates at high ambient temperature to respect the electrical components thermal capabilities of the turbine. Each manufacturer has a de-rating curve for his turbines. In this paper, Gamesa G80 wind turbine was used as an example to develop this model. The model is verified using historical temperature site data where, MCS is used to check that the proposed model matches the manufacturer de-rating curve. Finally, the wind power probability distribution is presented at different ambient temperature showing how the performance of wind energy system can vary significantly when the ambient temperature is taken into account. Hence, this model could provide more accuracy when wind energy systems are assessed in electrical power systems.

This paper is structured as follows: Firstly, the wind turbine speed modeling using various PDFs as well as the methods to estimate PDFs parameters are presented. Section 2. The optimization algorithms, EDO, AO and EO in addition to the problem formulation, are presented in Sect. 3. The classical wind energy modeling and wind power probability distribution is performed based on the historical data from Zaafarana in Egypt. Section 4. The novel model for wind energy considering the effect of ambient temperature is described in Sect. 5 where, the analysis is done based on Gamesa G80 wind turbine as a reference. Finally, In Sect. 6, the conclusion of the work is presented.

## Wind speed modeling

This section discusses the common PDFs used for wind speed modeling with a focus on the PDFs used in this paper. Moreover, Different methods, numerical and artificial intelligence methods are used to obtain the PDFs parameters.

### Probability distribution functions

#### Weibull probability function

The Weibull distribution is developed by Weibull^36^. It’s a two- parameter PDF where, the PDF, $\:{f}_{w}\left(v\right)\:$, and the cumulative function $\:{F}_{w}\left(v\right)$ are given by Eqs. (1–2) respectively. Weibull PDF is considered the most commonly used PDF for modelling the stochastic behaviour of wind energy.

1 $$\:{f}_{W}\left(v\right)\:=\:\frac{k}{{c}^{k}}\:{v}^{v-1}\:\text{e}\text{x}\text{p}(-({\frac{v}{c})}^{k}\:)$$

2 $$\:{F}_{W}\left(v\right)\:=1-\:\text{e}\text{x}\text{p}(-({\frac{v}{c})}^{k}\:)$$

Where, $\:v$ represents the wind speed, *k* and *c* are the shape and scale factors, respectively.

#### Lognormal probability function

The lognormal PDF, known as the Gaussian distribution, This probability function is named as the Galton distribution^37^ and with two parameters. Equation (3) represents the PDF, $\:{f}_{L}\left(v\right)$.

3 $$\:{f}_{L}\left(v\right)\:=\:\frac{1}{v{\upsigma\:}\sqrt{2\:\pi\:}}\:\:\text{e}\text{x}\text{p}(-(\frac{1}{2}\:\left({\frac{\text{ln}v-\mu\:}{\alpha\:{\upsigma\:}})}^{2}\right)$$

Where,, $\:v$ represents the wind speed, $\:\mu\:$ is mean value and σ is the standard deviation.

#### Gamma probability function

The gamma PDF is commonly used distribution due to its relation to exponential and normal distributions with two control parameters^38^. Equation (3) represents the PDF, $\:{f}_{G}\left(v\right)$.

4 $$\:{f}_{G}\left(v\right)\:=\:\frac{1}{{y}^{z}\:\varGamma\:\left(z\right)\:}\:\:{v}^{z-1}\:\text{e}\text{x}\text{p}(-\:\left(\frac{v}{y}\right))$$

Where, $\:v$ represents the wind speed, *y* and *z* are the scale and shape factors, respectively.

#### Mixed probability function

For enhanced representation, having mixed two or three PDFs was an approach provided in^34^ and it provided higher accuracy in modeling wind speed with lower errors compared to a single PDF. Equations (5–6) represent the mixed-two and mixed-three PDFs in a generic form.

5 $$\:{f}_{mixed-2}\:=\:{f}_{1}*{w}_{1}+\:{f}_{2}*{w}_{2}$$

6 $$\:{f}_{mixed-3}\:=\:{f}_{1}*{w}_{1}+\:{f}_{2}*{w}_{2}+\:{f}_{3}*{w}_{3}$$

Where, $\:{f}_{1}$, $\:{f}_{2}$ and $\:{f}_{3}$ are the first, second and third PDF respectively. $\:{w}_{1}$, $\:{w}_{2}$ and $\:{w}_{3}$ are the weights for each PDF function respectively. In case of mixed-2 function, the summation of weights, $\:{w}_{1}+\:{w}_{2}$, is 1. For mixed-3 function, the summation of weights, $\:{w}_{1}+\:{w}_{2}+{w}_{3}$, is 1.

### Methods for estimating PDF parameters

Several methods are used to estimate PDF parameters. In this part, two common categories will be discussed in brief; Numerical and AI methods^39^.

#### Numerical methods

This section provides an overview for the common numerical methods being used with Weibull PDF. Now AI methods have shown more accurate results compared to numerical methods.

##### Maximum likelihood method

Maximum likelihood method requires extensive numerical iteration. Shape and scale parameters can be calculated by using Eqs. (7–8).

7 $$\:k={[\:\frac{\sum\:_{i=1}^{n}{{v}_{i}}^{k}\text{ln}{v}_{i}}{\sum\:_{i=1}^{n}{{v}_{i}}^{k}}-\frac{\sum\:_{i=1}^{n}\text{ln}{v}_{i}}{n}\:]}^{-1}$$

8 $$\:c\:={\left(\frac{1}{n}\:\sum\:_{i=1}^{n}{{v}_{i}}^{k}\right)}^{\frac{1}{k}}$$

Where, $\:{v}_{i}$ is the wind speed in time step *i* and *n* is the number of non-zero points.

##### Modified maximum likelihood method

This method can be used when wind speed frequency distribution format is available. Equations (9–10) can be used to calculate the shape and scale parameters.

9 $$\:k={[\:\frac{\sum\:_{i=1}^{n}{{v}_{i}}^{k}\text{ln}{v}_{i}\:g\left({v}_{i}\right)}{\sum\:_{i=1}^{n}{{v}_{i}}^{k}f\left({v}_{i}\right)}-\frac{\sum\:_{i=1}^{n}\text{ln}{v}_{i}g\left({v}_{i}\right)}{f(v\:\ge\:0)}\:]}^{-1}$$

10 $$\:c\:={\left(\frac{1}{g(v\:\ge\:0)}\:\sum\:_{i=1}^{n}{{v}_{i}}^{k}\:g\left({v}_{i}\right)\right)}^{\frac{1}{k}}$$

Where, $\:{v}_{i}$ is the wind speed central to bin *i*, *n* the number of bins, $\:g\left({v}_{i}\right)\:$the frequency for wind speed ranging within bin i, and $\:g(v\:\ge\:0)$is the probability for wind speed equal to or exceeding zero.

##### Energy pattern factor

This method is based on averaging a data set of wind speeds. Energy pattern factor ($\:{E}_{PF}$), shape and scale factors are calculated as per Eqs. (11–13).

11 $$\:{E}_{PF}=\frac{\stackrel{-}{{v}^{3}}}{{\stackrel{-}{v}}^{3}}\:$$

12 $$\:k\:=1+\:\frac{3.69}{{{E}_{PF}}^{2}}$$

13 $$\:\:c=\:\:\frac{\stackrel{-}{v}}{\varGamma\:(1+\frac{1}{k})}\:$$

Where, $\:\stackrel{-}{{v}^{3}}$ is the mean of wind speed cubes, $\:\stackrel{-}{v}$ is the mean of wind speed.

##### Emperical method

This method is a subset of other method called the Moment method but using simplified formulas. Equations (13–14) can be used to calculate scale and shape factors respectively.

14 $$\:k\:=\:({\frac{\sigma\:}{v})}^{-1.086}$$

Where, $\:\sigma\:$ is the standard deviation of the wind data.

##### Graphical method

Using the principle of least squares, the graphic method fits a straight line to wind speed data; however, the time-series data must be divided into bins. Equation (15) represents the cumulative distribution function after applying double logarithmic transformation.

15 $$\:\text{ln}\{-\text{ln}(1-{F}_{W}\left(v\right))\}\:=\:k\text{ln}\left(v\right)-k\text{ln}\left(c\right)$$

#### Artificial intelligence method

**Fig. 1:**
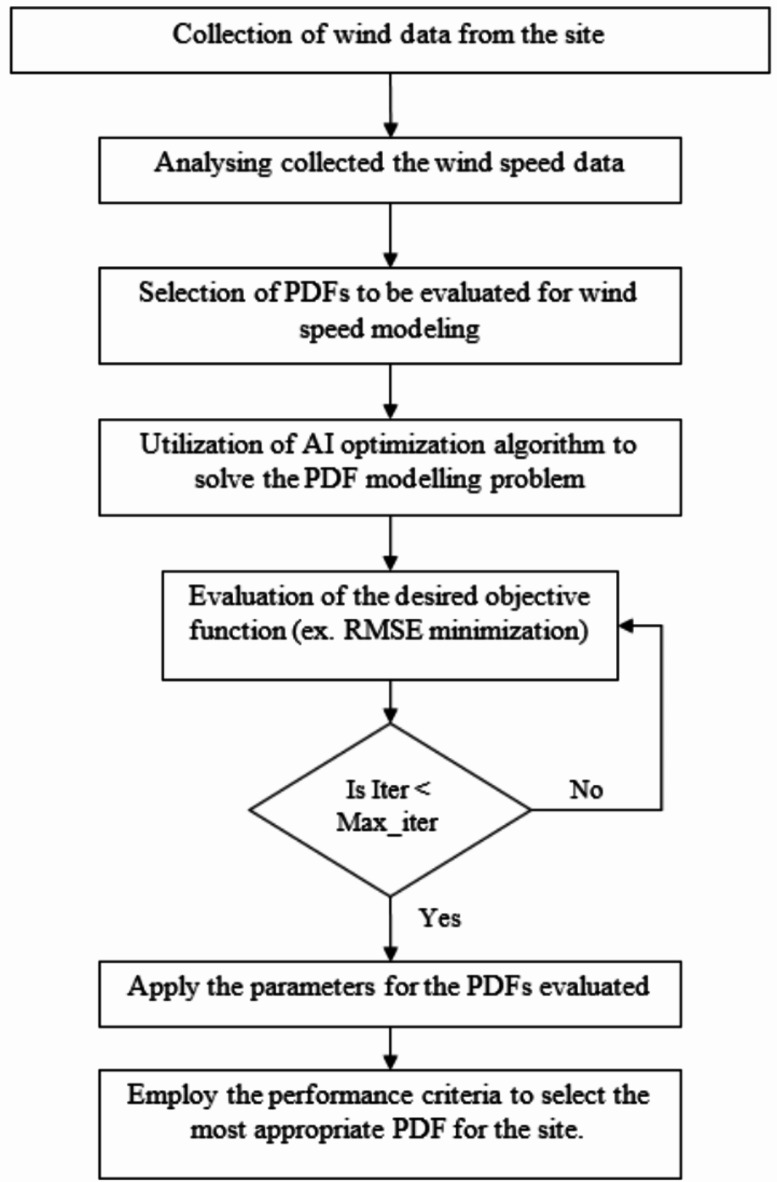
AI Optimization Method Flow Chart for Wind Speed Modeling.

Recently, finding the optimal parameters to model wind speed PDFs can be achieved by using artificial intelligence optimization algorithms as they showed more accuracy and reliable representation compared to other methods. The objective function is set as a test criterion, where, the choice of this objective function is the factor most crucial to the results. Figure 1 shows the flow chart for using AI optimization algorithms to obtain the best PDFs parameters for wind speed modeling.

In the next section, three AI optimization algorithms used in this paper are discussed in detail. Moreover, the objective function and evaluation criteria for selecting the suitable PDF are presented.

## Optimization algorithms and objective function

### Optimization algorithms

#### Exponential distribution optimizer

The EDO is a novel optimization algorithm introduced in 2023^40^. Statistical and severalbenchmark tests and engineering problems were used showing the excellence of EDO technique^40–42^. EDO takes its foundational principles from the theories of exponential distribution. this continuous probability distribution that is frequently used to simulate how long it takes for an event to occur. Moreover, it simulates the intervals of time between events that occur randomly and independently at a constant rate. An exponential random variable, *x*, associated with a rate parameter,$\:\:\lambda\:$. The relationship between these parameters can be expressed as PDF in Eq. (16)^41^.

16 $$\:f\left(x\right)=\left\{\begin{array}{c}\:\lambda\:{e}^{-\:\lambda\:x}\:\:\:\:\:\:\:\:\:\:\:\:\:\:x\ge\:0\\\:\:\:\:\:\:0\:\:\:\:\:\:\:\:\:\:\:\:\:\:otherwise\end{array}\right.$$

##### Memoryless feature

The “memoryless” feature of some statistical probability distributions is one of their distinctive features. This suggests that the likelihood that an upcoming event will occur is unaffected by previous occurrences. This memoryless quality is captured by the continuous exponential distribution, particularly when measuring the amount of time until an event occurs. When *x*, a random variable, follows the exponential distribution with this memoryless property, this means for any positive whole numbers s and t belonging to the series {0,1,2,. ∞} then the following comes true. Equations (17–20) explain the memoryless feature.

17 $$\:P\:(x\:>\:s+t\:|\:x\:\ge\:\:s)\:=\:P\:(x\:>\:t)\:\text{i}\text{f}\:t\:>\:0\:\&\:s\:>\:0$$

18 $$\:{\sigma\:}^{2}=\:\frac{1}{{\lambda\:}^{2}}$$

19 $$\:\lambda\:=\:1/\:\mu\:$$

20 $$\:\mu\:=\:0.5\:(\:{memoryless\:}_{i}^{time}+\:{Xguide}^{time}\:)$$

Where, $\:\sigma\:$ is the variance of exponentially random variable, $\:\lambda\:$ is the exponential rate, $\:\mu\:$ is the exponential mean and $\:{Xguide}^{time}$ is the guiding solution.

##### EDO exploitation

The exponential distribution model’s memoryless feature, exponential rate, typical variance, and average value are all utilized by the EDO’s exploitation part. A guiding solution also directs the search in the direction of the global peak. In EDO, a collection of randomly generated solutions is initially developed to resemble various exponential distribution patterns. There is a focus on a guiding solution calculated using Eq. (21).

21 $$\:{Xguide}^{time}=\frac{1}{3}*\:(\:{Xwinner\:}_{best}^{time1}+\:{Xwinner\:}_{best}^{time2}+{Xwinner\:}_{best}^{time3}\:)$$

In EDO, time replaces iterations, indicating the period until the next event in the exponential distribution, with the maximum iterations denoted as *Max_time*. The algorithm prioritizes a guiding solution over the current best solution to avoid convergence to a local maximum. A memoryless matrix is created to store the latest solutions and reflect the original population but is updated with new solutions at each step, independent of their past success. Solutions are categorized as either winners or losers. A solution is considered a winner if its efficiency surpasses that of its counterpart in the *Xwinners* group. If a solution is updated in both the *Xwinners* and memoryless matrices, it is classified as a winner; otherwise, it’s a loser. The exploitation phase is designed to update solutions according to the exponential distribution, focusing on improving winners using. The updated solution,$\:\:{V\:}_{i}^{time+1\:}$, is calculated using Eqs. (22–23).

22 $$\:\:{V\:}_{i}^{time+1\:}=\left\{\begin{array}{c}\:a.\left({memoryless\:}_{i}^{time}-{\sigma\:}^{2}\right)+b.{Xguide}^{time}\:\:if\:\:{Xwinners\:}_{i}^{time}={memoryless\:}_{i}^{time}\\\:b.\left({memoryless\:}_{i}^{time}-{\sigma\:}^{2}\right)+\:\text{log}\left(\varnothing\:\right){Xwinners\:}_{i}^{time}\:\:\:\:\:\:\:\:\:\:\:\:\:\:\:\:\:\:\:\:\:\:\:\:\:\:\:\:\:\:\:\:\:\:\:\:Otherwise\end{array}\right.$$

23 $$\:a={\left(f\right)}^{10},\:b={\left(f\right)}^{5}$$

Where, *a* and *b* are variables that can be changed.$\:\:\varnothing\:$ is a random number in the range [0, 1]. *f* is a random integer number in the range [-1, 1].

##### EDO exploration

The EDO exploration phase optimization model is developed using two random winners from the initial population who follow the exponential distribution, in this case $\:{X}_{winnder\:rand\:1}$ and $\:{X}_{winnder\:rand\:2}$ where, Eqs. (24–28) are used to update the solution.

24 $$\:{V\:}_{i}^{time+1\:}=\:{Xwinners\:}_{i}^{time}-\:{M}^{time}+(\left(\text{c}\:\:{Z}_{1}\right)+\left(1-\text{c}\right){Z}_{2})$$

25 $$\:{M}^{time}=\:\frac{1}{N}\:\sum\:_{i=1}^{N}{Xwinners\:}_{j,i}^{time}\:\:\:j=\text{1,2},\dots\:d$$

26 $$\:c=\:d\text{*}\:f\:,\:d=\:\frac{1-time}{Max\_time}$$

27 $$\:{Z}_{1}=M-{D}_{1}+\:{D}_{2}\:,\:{Z}_{2}=M-{D}_{2}+\:{D}_{1}$$

28 $$\:{D}_{1}=M-{X}_{winnder\:rand\:1}\:,\:{D}_{2}=M-{X}_{winnder\:rand\:2}$$

Where, $\:{M}^{time}$ is the of all solutions average obtained from initial population, $\:{Z}_{1}$ and $\:{Z}_{2}$ vectors towards the contemporary solution, *c* is refined parameter representing the information exchange ratio between$\:\:{Z}_{1}$ and $\:{Z}_{2}$ vectors, *N* is the population size, *time* refers to iteration number and $\:Max\_time$ is the total number of iterations, *d* is the dimension of the optimization problem. $\:{D}_{1}$ and $\:{D}_{2}$ identify the distance between the average solution and the ’winners’ randomly picked from the initial population.

Sensitivity analysis for EDO has been done through changing the algorithm parameters by 10% in^43^. The results showed that with the change of EDO parameters the sum square error is minimized keeping the same conclusions.

**Fig. 2:**
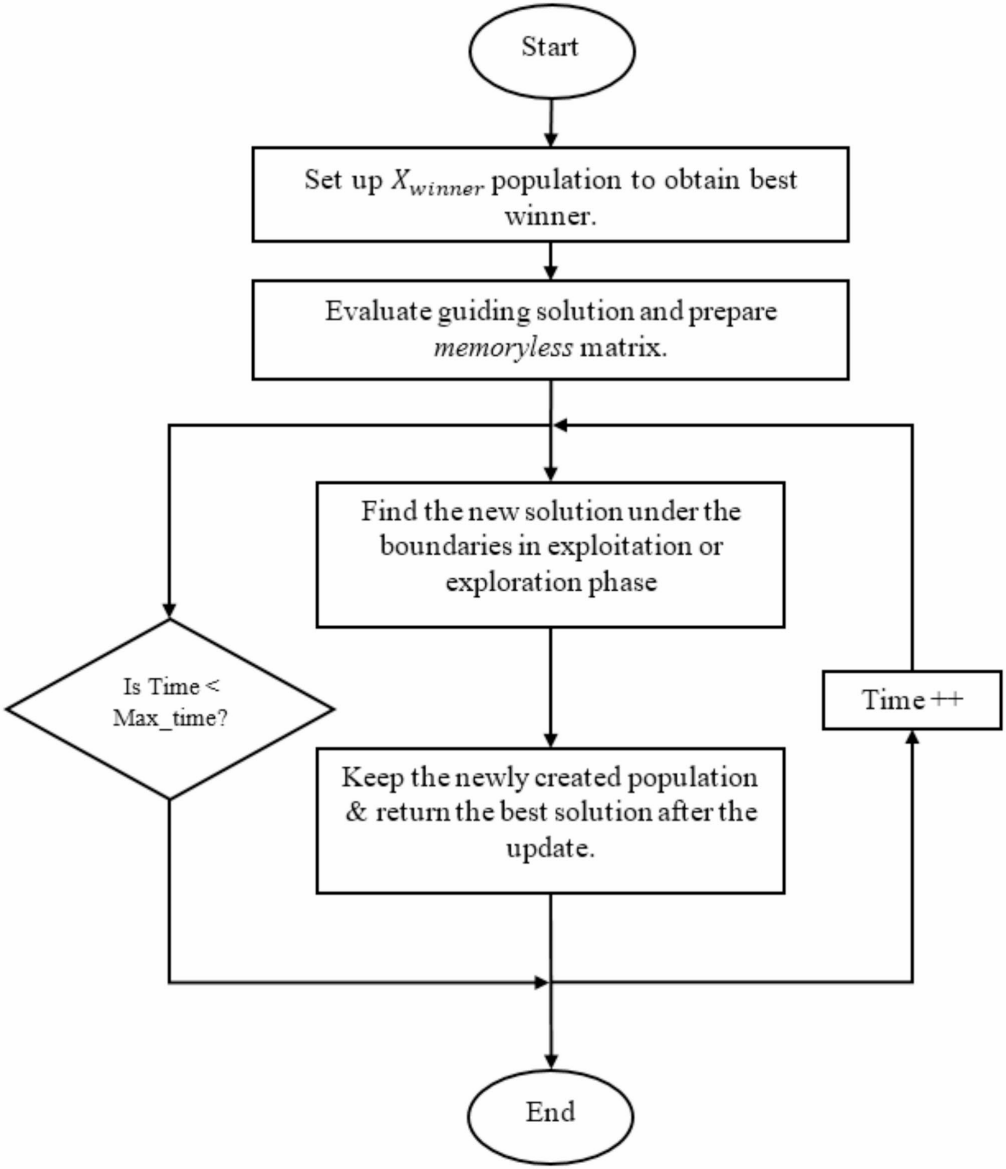
EDO Flowchart.

Figure 2 shows the flow chart for the EDO algorithm^44^.

#### Aquila optimizer

This algorithm was motivated by the Aquila’s natural behaviours while hunting its prey. The algorithm was inspired by the four primary hunting strategies used by Aquila, including high soar with a vertical stoop, contour flight with a short glide, low fly with a gradual descent, and walking and grabbing prey. There are four stages from exploration to exploitation of AO algorithm that show the methodology of the technique. Where, the optimization algorithm starts with a population of potential solutions (X) as follow^45^.

##### Stage 1: expanded exploration x_1_

Expanded Exploration fundamentally brings to light the principal hunting approach employed by Aquila, which is the high soar with vertical stoop in which the algorithms try to figure out the search apace from the high ascend, and the same is mathematically expressed using Eq. (29).

29 $$\:{x}_{1}\left(\text{t}+1\right)=\left({x}_{best}\left(\text{t}\right)\text{*}\:\frac{1-t}{T}\right)+({x}_{M}\left(\text{t}\right)-{x}_{best}\left(\text{t}\right)*rand)$$

Where, $\:{x}_{1}\left(\text{t}+1\right)$ is the t-next step solution which is concluded from 1st search approach $\:{x}_{1}$, $\:{x}_{best}\left(\text{t}\right)$

is the best obtained result, *rand* is random number in [0,1] interval, t and T are the current step and the maximum number of iterations. $\:{x}_{M}\left(\text{t}\right)$ is the location mean value of current solution.

##### Stage 2: narrowed exploration x_2_

This stage is the second hunting technique carried out by Aquila that is the contour flight with short glide is projected, wherein Aquila arranges the land thereby circling around the target prey to attack. Such behavior of Aquila is represented by Eqs. (30–31).

30 $$\:{x}_{2}\left(\text{t}+1\right)=\left({x}_{best}\left(\text{t}\right)\text{*}Levy\left(D\right)\right)+({x}_{R}\left(\text{t}\right)+(y-z\left)*rand\right)$$

31 $$\:Levy\left(D\right)=s*\:\:\frac{u*\sigma\:}{{\left|v\right|}^{\frac{1}{\beta\:}}}$$

Where, $\:{x}_{2}\left(\text{t}+1\right)\:$solution of the next iteration of *t*, $\:{x}_{R}\left(\text{t}\right)\:$represents random solution belonging to population size, *y* and *z* are the spiral shape in the search, *Levy (D)* is the Lévy flight distribution function for D which is the dimension space. *s* is the constant value of 0.01, *u & v* are random numbers in [0,1] interval, $\:\beta\:$ is the constant value of 0.5 and $\:\sigma\:$ is a parameter for Lévy flight distribution function.

##### Stage 3: expanded explolitation x_3_

The concept behind the third hunting mechanism is outlined as low flying with a slow descent attack in which Aquila progressively plunges into the targeted space and moves closer to his target to strike. Equation (32) appropriately describes Aquila’s activity.

32 $$\:{x}_{3}\left(\text{t}+1\right)=\left({x}_{best}\left(\text{t}\right)\text{*}{x}_{M}\left(\text{t}\right)\right)\alpha\:-rand+\left(\left(UB-LB\right)*rand+LB\right)*\delta\:$$

Where, $\:UB$ and $\:LB$ are upper and lower boundaries of the optimization problem,$\:\:\alpha\:$ and $\:{\updelta\:}$ are the fixed exploitation adjustment parameters and taken as 0.1.

##### Stage 4: narrowed explolitation x_4_

It’s the final step, the idea is taken from the last attacking way of Aquila widely known as walk and grab attack. Equation (33) is mathematically represents this stage.

33 $$\:{x}_{4}\left(\text{t}+1\right)=\left({x}_{best}\left(\text{t}\right)\text{*}QF\right)-\:\left(X\left(\text{t}\right)\text{*}{G}_{1}*rand\right)-\:{(G}_{2}\:\text{*}Levy\left(D\right) + rand\:\text{*}\:{G}_{1})$$

**Fig. 3:**
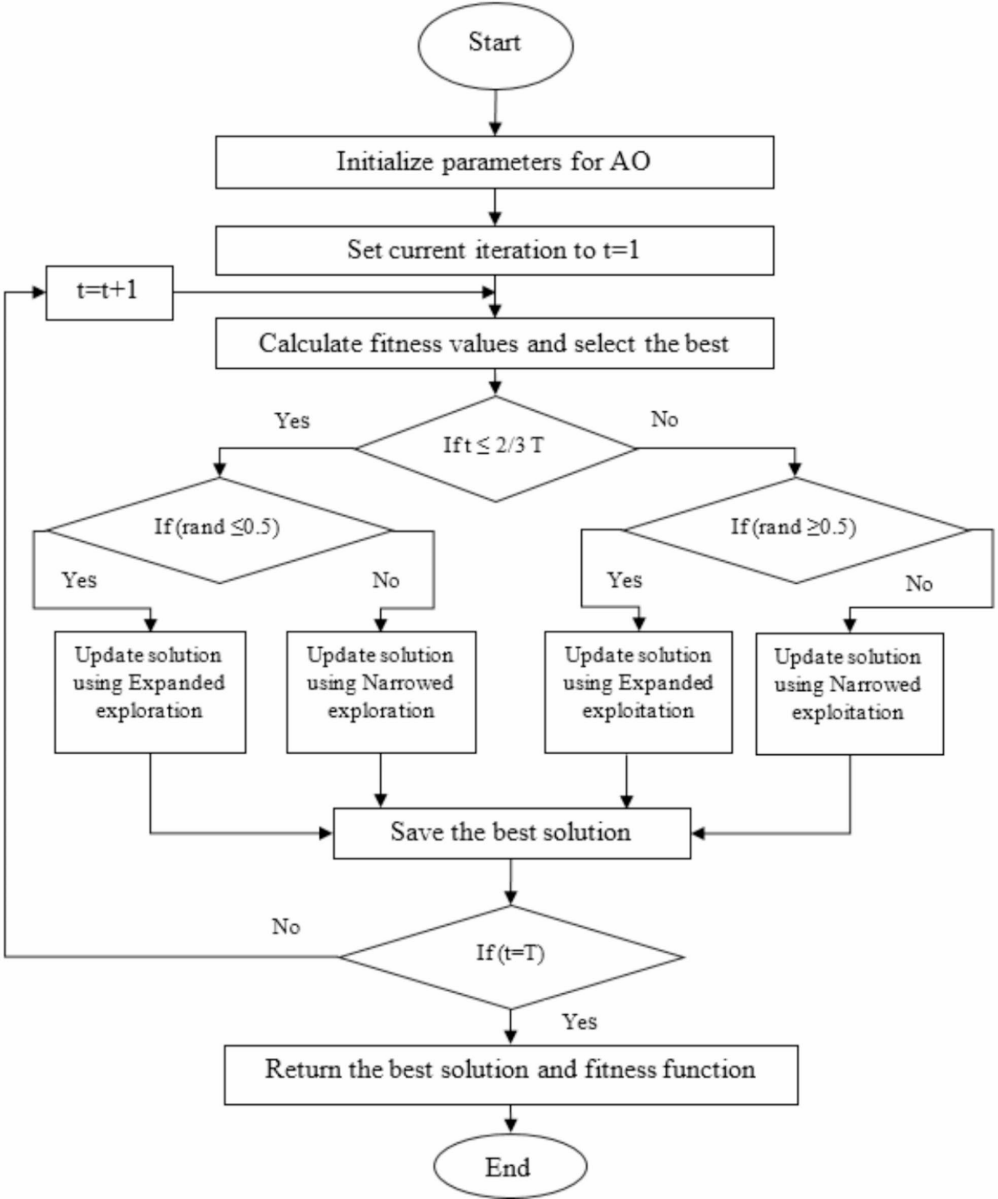
AO Flow Chart.

Where, $\:QF$ is the quality function used to balance the search way, $\:{G}_{1}$and $\:{G}_{2}$ represents the various motions and flight slope of AO respectively. Figure 3 shows the flow chart for the AO algorithm^46^.

#### Equilibrium optimizer

The Equilibrium optimizer is one of recent optimization approach based on the control volume mass balance, which is used to determine both dynamic and equilibrium states. In the EO approach, each particle (solution) and its concentration (position) serve as search agents. In this strategy, search agents adjust their concentration (position) at random in reference to certain skilled particles, known as equilibrium candidates, in order to attain optimal outcomes^47^.

A first-order ordinary differential equation defines the general mass balancing equation in which the change in mass over time is equal to the amount of mass that enters the system plus the amount being created within minus the amount exiting the system, as described in Eqs. (34–35).

34 $$\:V\frac{dC}{dt}=Q\:{C}_{eq}-QC+\:G$$

35 $$\:\frac{dC}{\lambda\:{C}_{eq}\:-\lambda\:C\:+\:\frac{G}{V}\:}=dt$$

By integrating both sides the resulting dynamic mass balance equation is expressed in Eq. (36) and the exponential term *F* can be calculated by Eq. (37).

36 $$\:C={C}_{eq}+\left({C}_{0}-\:{C}_{eq}\right)F+\:\frac{G}{\lambda\:V}(1-F)$$

37 $$\:F={e}^{-\lambda\:(t-{t}_{0})}$$

where, *C* is the concentration inside the control volume, the term, $\:V\frac{dC}{dt}$, is the rate of change of mass in the control volume, *Q* is the volumetric flow rate into and out of the control volume, $\:{C}_{eq}$ represents the concentration at an equilibrium state where there is no generation inside the control volume, and G is the mass generation rate inside the control volume. When $\:V\frac{dC}{dt}$ reaches zero accordingly the equilibrium state is reached. *λ* is the turnover rate. $\:{t}_{0}\:$and $\:{C}_{0}$ are the initial start time and concentration dependent on the integration interval.

The main steps of the EO can be summarized as follows:

1. Initialization of particles and function evaluation where initial concentration $\:{C}_{i}^{\:initial}$ is randomly evaluated
2. Preparation of equilibrium pool $\:{C}_{eq,pool}$ and candidates
3. Calculation of the exponential term (*F*).
4. Calculation of generation rate (*G*).
5. Updating the EO rule with the new concentration till the maximum number of iterations is reached.

**Fig. 4:**
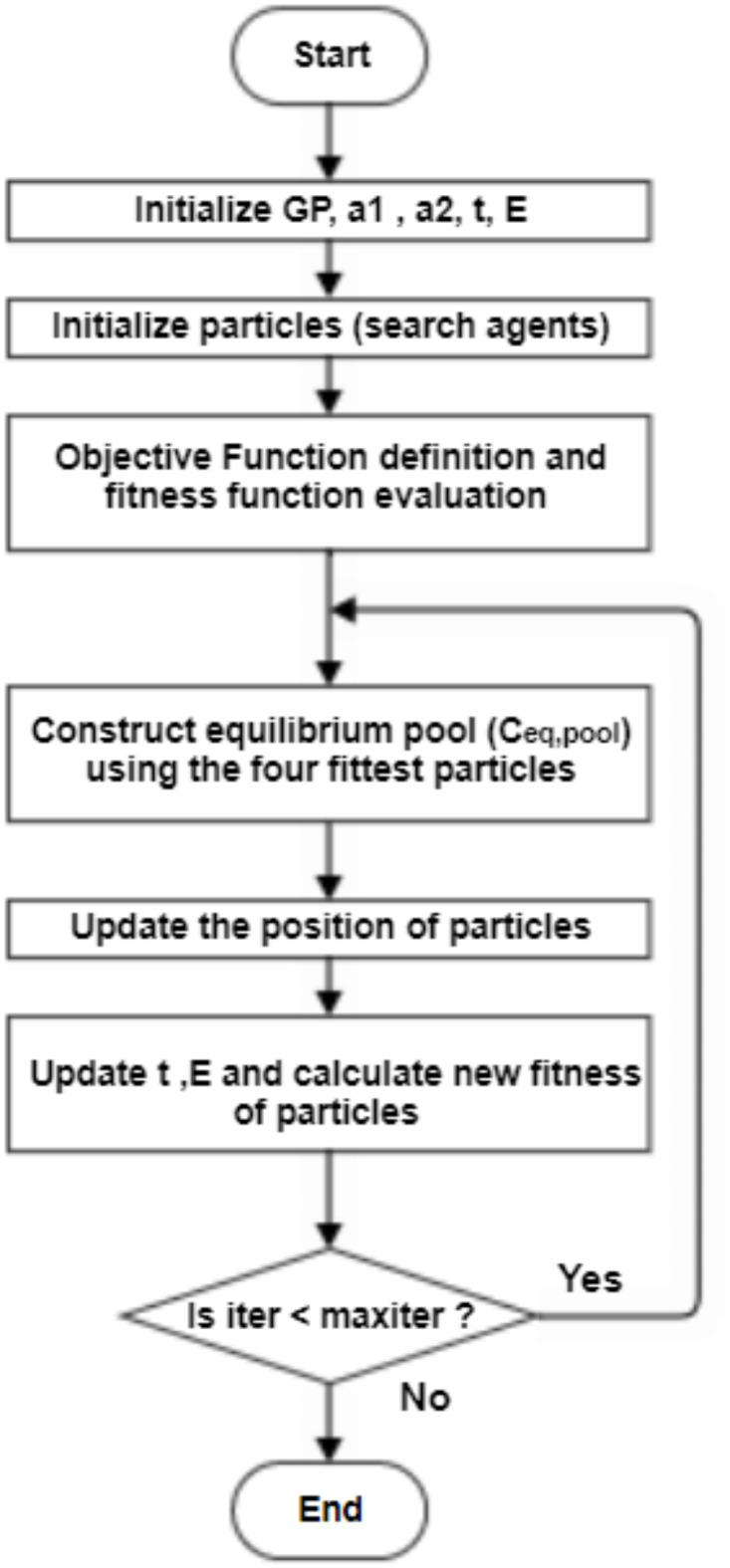
EO flow chart.

Figure 4 illustrates the flow chart of EO technique^48^.

### Objective function

This part covers the problem formulation used in the optimization problem to model wind speed. Equation (38) represents the objective function to minimize the *RMSE* between the PDF fitting curve and wind speed frequency distribution.

38 $$\:RMSE=\sqrt{\frac{1}{2}\:\sum\:_{i=1}^{k}{({y}_{i}-{x}_{i})}^{2}}$$

Where, *k* is the number of wind speed classes, $\:{y}_{i}$ represents the $\:{i}^{th}$ wind speed from real data and $\:{x}_{i}$ represents the anticipated $\:{i}^{th}$ wind speed.

## Wind energy modeling

In this section, the classical wind speed modeling including mixed PDFs, used in previous studies, are presented. A comparision between the various PDFs is done to select an appropriate PDF to introduce the novel wind energy model. Afterwards, The novel wind energy modeling considering the impact of ambient temperature is discussed. It’s important to mention that based on the wind speed modeling, Weibull PDF is used to present the novel wind energy model.

### Wind speed modeling

Three years of wind speed readings have been used from Zaafrana region in Egypt to model the wind speed. EDO, AO, and EO algorithms are used to obtain the distribution parameters for each PDF. The objective is to minimize the RMSE mentioned in Eq. (38). Moreover, Coefficient of Correlation $\:{R}^{2}$ expressed in Eq. (39) and *RMSE* are used as judging criteria.

39 $$\:{R}^{2}=\:\frac{\sum\:_{i=1}^{k}{({y}_{i}-{v}_{av})}^{2}-\sum\:_{i=1}^{k}{({x}_{i}-{v}_{av})}^{2}}{\sum\:_{i=1}^{k}{({y}_{i}-{v}_{av})}^{2}}$$

Where, *k* is the number of wind speed classes, $\:{y}_{i}$ represents the $\:{i}^{th}$ wind speed from real data and $\:{x}_{i}$ represents the anticipated $\:{i}^{th}$ wind speed,$\:\:{v}_{av}$ is the average wind speed.

**Fig. 5:**
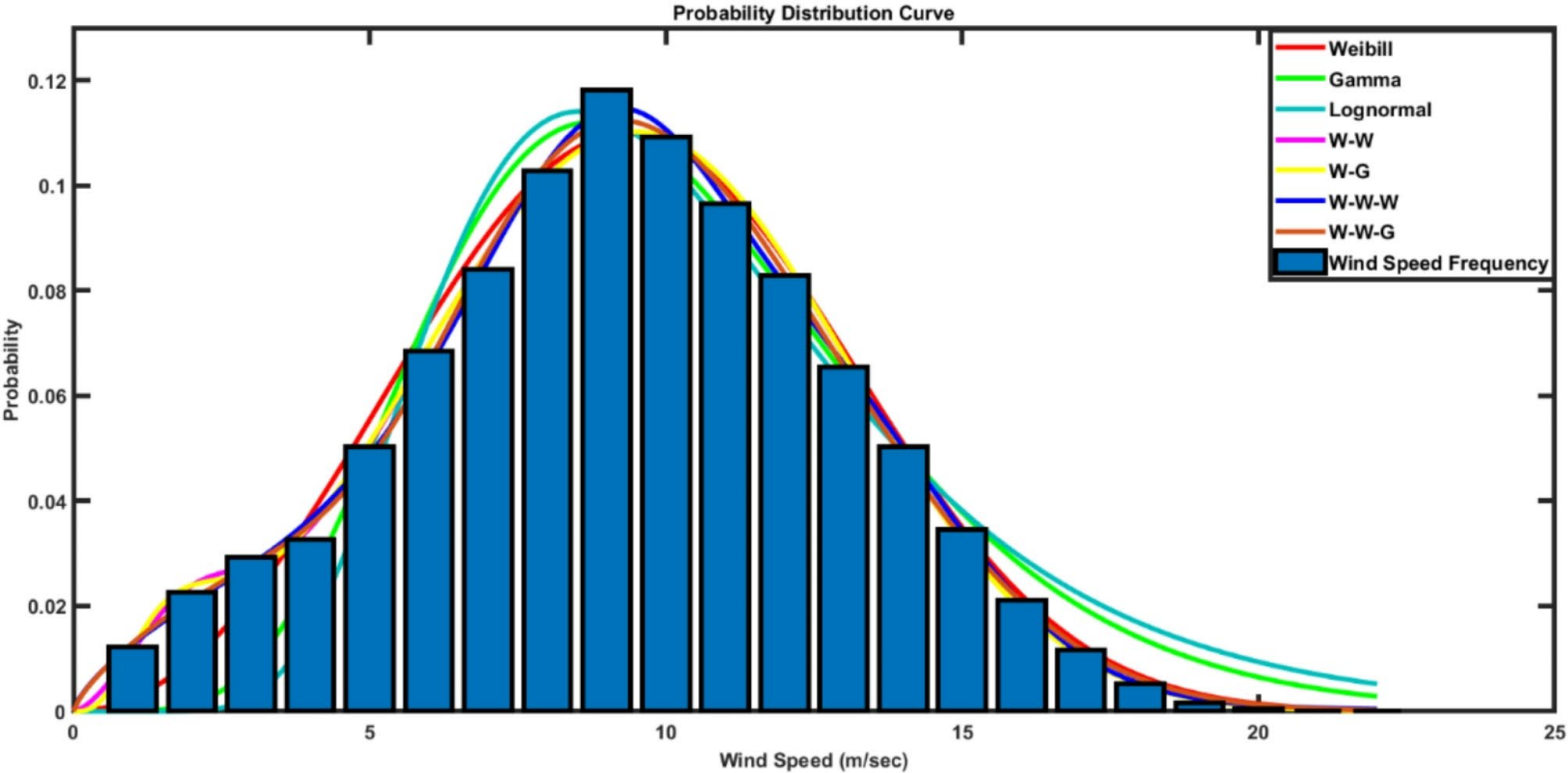
Wind Speed Modeling.

Figure 5 shows the fitting of the original wind speed distribution against different PDFs. In terms of single PDF, Weibull and Gamma PDFs showed better representation than lognormal function. Accordingly, Mixed two and Mixed three PDFs using Weibull and Gamma are assessed to better representation. Equations (40–43) show the equations used for mixed PDFs.

40 $$\:{f}_{W-W}\:=\:{f}_{{Weibull}_{1}}*{w}_{1}+\:{f}_{{Weibull}_{2}}*(1-{w}_{1})$$

41 $$\:{f}_{W-G}\:=\:{f}_{Weibull}*{w}_{1}+\:{f}_{Gamma}*(1-{w}_{1})$$

42 $$\:{f}_{W-W-W}\:=\:{f}_{{Weibull}_{1}}*{w}_{1}+\:{f}_{{Weibull}_{2}}*{w}_{2}++\:{f}_{{Weibull}_{3}}*{(1-{w}_{1}-w}_{2})\:$$

43 $$\:{f}_{W-W-G}\:=\:{f}_{{Weibull}_{1}}*{w}_{1}+\:{f}_{{Weibull}_{2}}*{w}_{2}++\:{f}_{Gamma}*{(1-{w}_{1}-w}_{2})$$

Table 1 shows the parameters obtained from each optimization algorithm.

**Table 1:** PDFs parameters.

PDF	AO	EDO	EO
***Weibull***	$\:k$= 2.975 $\:c$= 10.701	$\:k$= 2.995 $\:c$= 10.6917	$\:k$= 2.9958 $\:c$= 10.6916
***Gamma***	y = 1.387 z = 7.3213	y = 1.408 z = 7.218	y = 1.408 z = 7.2179
***Lognormal***	$\:\mu\:$=2.278 σ = 0.384	$\:\mu\:$= 2.2838 σ = 0.3784	$\:\mu\:$=2.2891 σ = 0.38104
***Mixed W-W***	$\:{k}_{1}$= 3.7209 $\:{c}_{1}$= 10.7419 $\:{k}_{2}$=1.866 $\:{c}_{2}$=10.270 $\:{w}_{1}$=0.6605	$\:{k}_{1}$= 3.147 $\:{c}_{1}$=10.6915 $\:{k}_{2}$= 2.497 $\:{c}_{2}$=2.3749 $\:{w}_{1}$=0.9617	$\:{k}_{1}$= 3.146 $\:{c}_{1}$=10.6916 $\:{k}_{2}$= 2.499 $\:{c}_{2}$=2.3725 $\:{w}_{1}$=0.9618
***Mixed W-G***	$\:k$= 3.053 $\:c$= 10.674 $\:y$=5.8428 $\:z$=8.743 $\:{w}_{1}$=0.918	$\:k$= 3.1345 $\:c$= 10.69 $\:y$= 0.4389 $\:z$=5.06179 $\:{w}_{1}$= 0.962	$\:k$= 3.15 $\:c$= 10.696 $\:y$= 0.4535 $\:z$=4.87 $\:{w}_{1}$=0.9625
***Mixed W-W-W***	$\:{k}_{1}$= 1.5045 $\:{c}_{1}$= 9 $\:{k}_{2}$= 4.708 $\:{c}_{2}$= 9 $\:{k}_{3}$= 3.58 $\:{c}_{3}$=11.39 $\:{w}_{1}$=0.2145 $\:{w}_{2}$=0.145	$\:{k}_{1}$= 4.99 $\:{c}_{1}$= 8.998 $\:{k}_{2}$= 4.149 $\:{c}_{2}$=12 $\:{k}_{3}$= 1.7283 $\:{c}_{3}$=8.34 $\:{w}_{1}$=0.206 $\:{w}_{2}$=0.5	$\:{k}_{1}$= 4.99 $\:{c}_{1}$= 8.997 $\:{k}_{2}$= 1.7227 $\:{c}_{2}$=8 $\:{k}_{3}$= 4.07 $\:{c}_{3}$=12 $\:{w}_{1}$=0.204 $\:{w}_{2}$= 0.272
***Mixed W-W-G***	$\:{k}_{1}$=1.888 $\:{c}_{1}$=8.8208 $\:{k}_{2}$= 4.1026 $\:{c}_{2}$=9.42 $\:y$= 0.816 $\:z$=13.2138 $\:{w}_{1}$=0.3816 $\:{w}_{2}$=0.104	$\:{k}_{1}$= 3.708 $\:{c}_{1}$= 11.967 $\:{k}_{2}$= 1.8417 $\:{c}_{2}$=7.345 $\:y$= 0.7945 $\:z$=12.48 $\:{w}_{1}$= 0.332 $\:{w}_{2}$= 0.257	$\:{k}_{1}$= 3.5928 $\:{c}_{1}$= 11.903 $\:{k}_{2}$= 1.754 $\:{c}_{2}$= 7 $\:y$= 0.7356 $\:z$= 13.207 $\:{w}_{1}$= 0.406 $\:{w}_{2}$= 0.229

These parameters are used to evaluate various wind speed PDFs. Table 2 shows the *RMSE* and the Coefficient of Correlation obtained for the evaluated PDFs.

**Table 2:** RMSE and the coefficient of correlation for PDFs.

PDF	Evaluation Criteria	AO	EDO	EO
***Weibull***	*RMSE*	0.00507	0.00506	0.00506
	$\:{R}^{2}$	0.9832	0.9836	0.9836
***Gamma***	*RMSE*	0.00892	0.00891	0.00891
	$\:{R}^{2}$	0.9489	0.9482	0.94823
***Lognormal***	*RMSE*	0.01139	0.01133	0.01131
	$\:{R}^{2}$	0.915	0.918	0.916
***Mixed W-W***	*RMSE*	0.00391	0.002618	0.002618
	$\:{R}^{2}$	0.9897	0.9954	0.9954
***Mixed W-G***	*RMSE*	0.00498	0.0027	0.00269
	$\:{R}^{2}$	0.98407	0.995	0.9951
***Mixed W-W-W***	*RMSE*	0.0023	0.00172	0.00168
	$\:{R}^{2}$	0.9962	0.998	0.998
***Mixed W-W-G***	*RMSE*	0.00293	0.002094	0.00203
	$\:{R}^{2}$	0.994	0.997	0.997

The results show that the Weibull PDF provides 3% and 6.8% higher Coefficient of Correlation as well as 56% and 64% lower *RMSE* compared to Gamma and Lognormal PDFs respectively. Moreover, the mixed three PDFs provides 1.5% and 0.5% higher Coefficient of Correlation as well as 55% and 42% lower *RMSE* compared to single and mixed two PDFs respectively. EDO and EO show 25% and 27% reduced *RMSE* compared to AO technique.

### Classical wind energy modeling

The output equation used to represent the output power from a wind turbine as a function of wind speed is described in Eq. (44):

44 $$\:{P}_{WT}=\left\{\begin{array}{c}0\:\:\:\:\:\:\:\:\:\:\:\:\:\:\:\:\:\:v<{v}_{cut-in}\\\:\:{P}_{r}\:(\frac{v-{v}_{cut-in}}{{v}_{r}-\:{v}_{cut-in}})\:\:\:\:{v}_{cut-in}\:\le\:v\:\le\:\:{v}_{r}\\\:\:{P}_{r}\:\:\:\:\:\:\:\:\:\:\:\:\:\:\:{v}_{r}\:\le\:v\:\le\:\:{v}_{cut-out}\end{array}\right.$$

Where, $\:\:{P}_{r}$is the rated power of the wind turbine, $\:{v}_{cut-in}$ is the wind turbine cut-in speed, $\:v$ is the wind speed, $\:{v}_{r}$ is the wind turbine rated speed, $\:{v}_{cut-out}$ is the wind turbine cut-out speed.

After comparing various methods for wind speed modeling, we have chosen to use the Weibull distribution to discuss the influence of temperature on wind power probability distribution which will be discussed in the next section. The results of this analysis can be generalized to other distribution curves as well. The wind power probability distribution using Weibull curve for different wind speeds$\:\:{f}_{w}\left(P\right)$, has discrete values for zero power and rated power. These discrete probabilities are expressed in Eqs. (45–46). The wind power probability is continuous between $\:{v}_{cut-in}$ and $\:{v}_{r}$ as expressed in Eq. (47)^49^.

45 $$\:{f}_{w}\left(P\right)\left\{\:{P}_{wind}=0\right\}=1-\text{exp}\left(-{\frac{{v}_{cut-in}}{c})}^{k}\right)+\:\text{exp}\left(-{\frac{{v}_{cut-out}}{c})}^{k}\right)$$

46 $$\:{f}_{w}\left(P\right)\left\{\:{P}_{wind}=\:{P}_{r}\right\}=\:\text{exp}\left(-{\frac{{v}_{r}}{c})}^{k}\right)-\:\text{exp}\left(-{\frac{{v}_{cut-out}}{c})}^{k}\right)$$

47 $$\:{f}_{w}\left(P\right)=\:\frac{k\:({v}_{r}-{v}_{cut-in})}{{c}^{k}*Pr}\:{({v}_{cut-in}+\frac{p}{Pr}\left({v}_{r}-{v}_{cut-in}\right))}^{k-1}*\text{e}\text{x}\text{p}(-{\left(\frac{{v}_{cut-in}+\frac{P}{Pr}\left({v}_{r}-{v}_{cut-in}\right)}{c}\right)}^{k})$$

### Novel wind energy modeling

Environmental conditions play an important role in electrical networks. One of these conditions is the ambient temperature effect which plays an important role in the performance of renewable energies. In this section, a novel wind energy modeling is presented to reflect the impact of the ambient temperature on the performance of wind turbine.

**Fig. 6:**
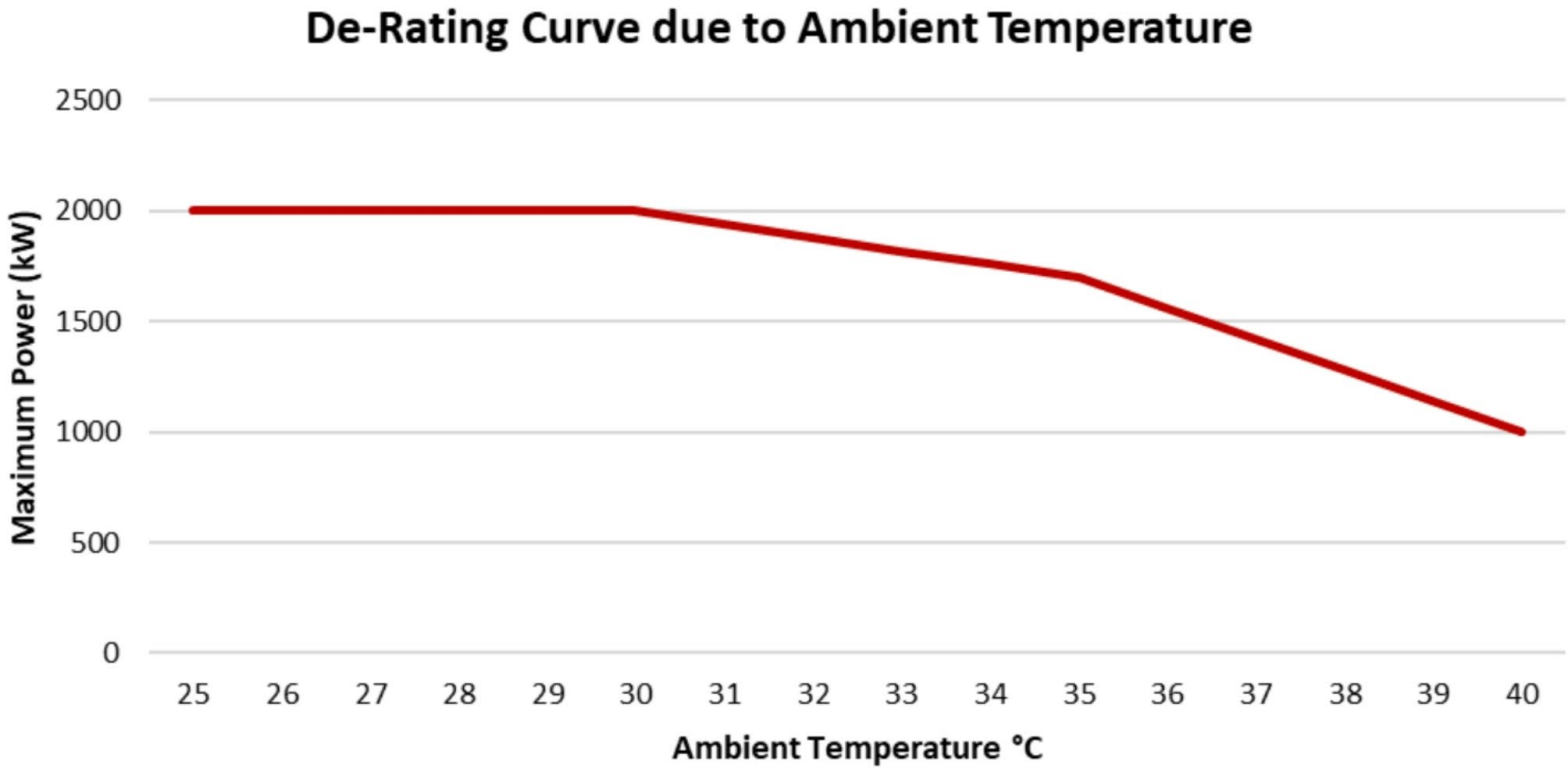
G80 De-Rating Curve due to Ambient Temperature.

The output power of the wind turbine is affected by ambient temperature. Where, according to the ambient temperature the power output is de-rated. Thus, the maximum power that can be generated from a wind turbine,$\:\:{P}_{max}$, will be functioning in the ambient temperature$\:\:{T}_{amb}$. In practice, this relation is introduced as a de-rating curve for active power when the ambient temperature increases above a specific value set by each manufacturer the power is reduced as the internal temperature of the turbine increases. When of the ambient temperature, the air density decreases thus the cooling system becomes insufficient to reduce the internal temperature so the power is de-rated. So for each ambient temperature, there is a maximum power that can be generated. Figure 6 shows the de-rating curve for G80 wind turbine that is used in the proposed model in Section *V*.

According to the figure, below 30 °C, the maximum power that could be generated from the wind turbine is its rated power, 2000 kW. With the increase of the ambient temperature the maximum power decreases till a certain maximum ambient temperature is reached, in this case is 40 °C where above this temperature the wind turbine is switched off to avoid any impact on the electrical components.

To address impact of the the ambient temperature, Eqs. (48–50) explains the novel model in a generic form, where due to ambient temperature the output rated power,$\:\:{P}_{r}$, can’t be guaranteed. Hence, the model is introduced considering new terminologies compared to the classical model presented in the previous section. where, $\:{P}_{r}$ will be replaced by $\:\:{P}_{max}$ and $\:{v}_{r}$ will be replaced by $\:{v}_{max}\:$.

$\:{v}_{max}$ is the wind speed corresponding to the maximum output power at specific ambient temperature. Where, if the wind speed increase but the ambient temperature still doesn’t change accordingly the output power won’t increase.

48 $$\:{P}_{WT}=\left\{\begin{array}{c}0\:\:\:\:\:\:\:\:\:\:\:\:\:\:\:\:\:\:v<{v}_{cut-in}\\\:\text{}{P}_{{max}}\:(\frac{v-{v}_{cut-in}}{{v}_{max}-\:{v}_{cut-in}})\:\:\:\:{v}_{cut-in}\:\le\:v\:\le\:\:{v}_{max}\\\:\:\:{P}_{max}\:\:\:\:\:\:\:\:\:\:\:\:\:\:\:{v}_{max}\:\le\:v\:\le\:\:{v}_{cut-out}\end{array}\right.$$

49 $$\:{v}_{max}=\:{v}_{cut-in}+\left(\frac{\:{P}_{max}}{{P}_{r}}\:\text{*}\left({v}_{r}-\:{v}_{cut-in}\right)\right)$$

50 $$\:{P}_{max}=\left\{\begin{array}{c}{P}_{r}\:\:\:\:\:\:\:\:\:\:\:\:\:\:\:\:\:\:\:{T}_{amb}\le\:{T}_{d}\\\:{P}_{r}-m({T}_{amb}-\:{T}_{d})\:\:\:\:\:{T}_{d}\le\:{T}_{amb}\:\le\:\:{T}_{off}\\\:\:\:0\:\:\:\:\:\:\:\:\:\:\:\:\:\:\:{T}_{d}>{T}_{off}\:\end{array}\right.$$

Where, $\:{T}_{d}$ is the temperature at which the de-rating curve starts. $\:m$ is the slope of the de-rating curve from obtained from the wind turbine manufacturer. $\:{T}_{off}$ is the maximum temperature at which the turbine can operate and beyond this temperature, the wind turbine will shut down to avoid any impact on its components. Below $\:{T}_{d}$, the maximum power that could be generated is the wind turbine rated power, and $\:{v}_{max}$ is the rated wind speed needed to generate this power.

It can be observed that the ambient temperature has a significant effect on the output power from a wind turbine. Accordingly, this has to be reflected on the wind power probability distribution. Based on the wind speed modeling results, the Weibull PDF can be used as a base for the wind power probability distribution.

The wind power probability for different wind speeds,$\:\:{f}_{w}\left(P\right)$, will be reformulated considering the de-rating effect due to ambient temperature. The wind power probability has discrete values for zero power which will be similar to Eq. (45) while for maximum output power, Eq. (51) can be used. The wind power probability is continuous between $\:{v}_{cut-in}$ and $\:{v}_{max}$ as expressed in Eq. (52). According to the ambient temperature the value for $\:{P}_{max}$ and $\:{v}_{max}$ can be calculated to determine the wind power probability distribution. Where, without any change in the ambient temperature, $\:{P}_{max}$ is maintained by the wind turbine controller to respect the wind turbine limitations defined by the manufacturer.

51 $$\:{f}_{w}\left(P\right)\left\{\:{P}_{wind}=\:{P}_{max}\right\}=\:\text{exp}\left(-{\frac{{v}_{max}}{c})}^{k}\right)-\:\text{exp}\left(-{\frac{{v}_{cut-out}}{c})}^{k}\right)$$

52 $$\:{f}_{w}\left(P\right)=\:\frac{k\:({v}_{max}-{v}_{cut-in})}{{c}^{k}*{P}_{max}}\:{({v}_{cut-in}+\frac{P}{{P}_{max}}\left({v}_{max}-{v}_{cut-in}\right))}^{k-1}*\text{e}\text{x}\text{p}(-{\left(\frac{{v}_{cut-in}+\frac{P}{{P}_{max}}\left({v}_{max}-{v}_{cut-in}\right)}{c}\right)}^{k}$$

In the next section, the simulation results for the classical and novel wind energy models are presented.

## Simulation & results

In this section, First, the simulation results for the classical wind energy model are presented including the wind energy probability distribution. Second, the results of the novel proposed model are presented. Finally, the difference between the classical and novel models are presented.

### Classical wind energy modeling results

**Fig. 7:**
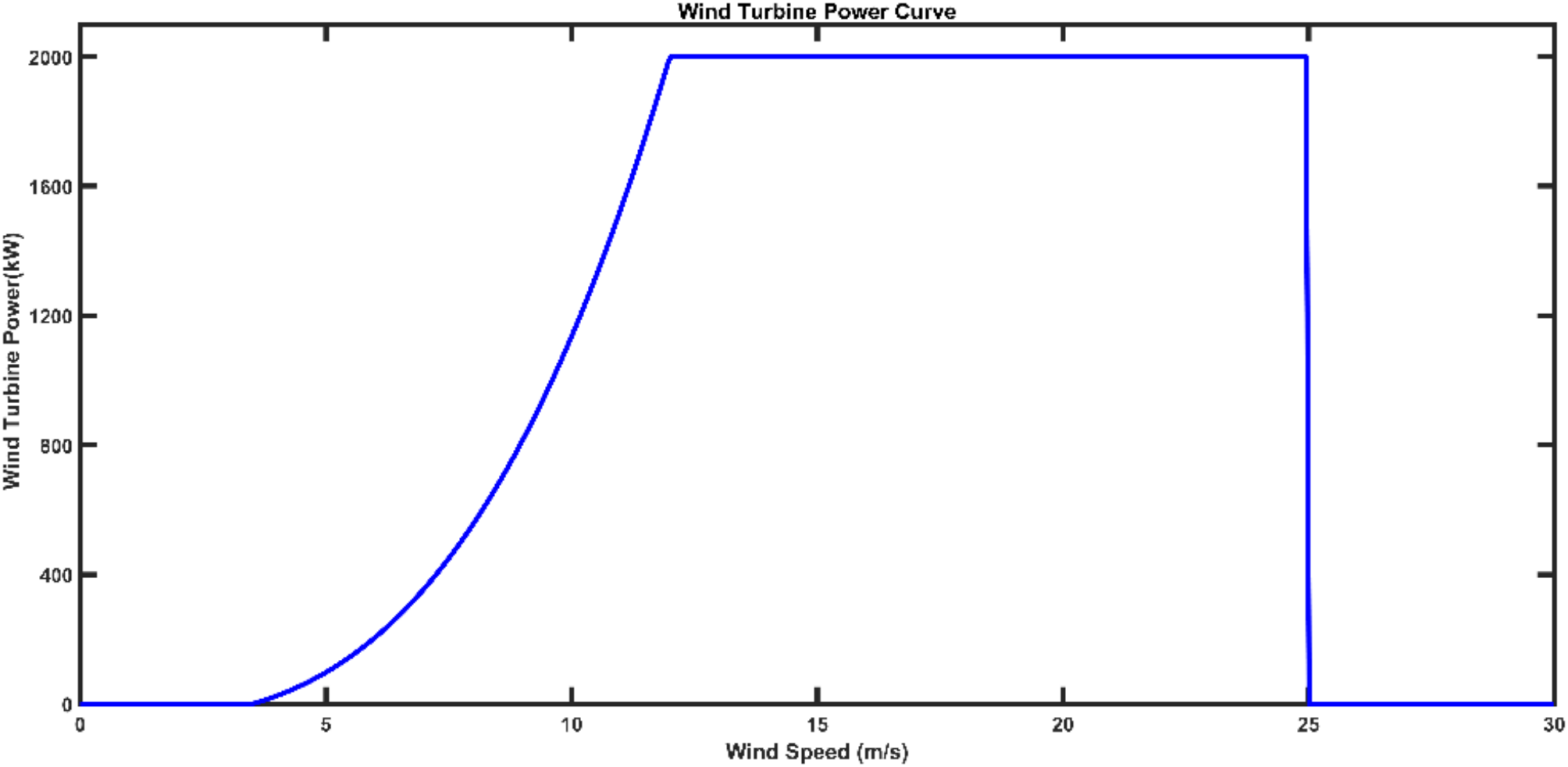
Wind Tubrine Power Curve.

The work presented considers a 2000 kW turbine with $\:{v}_{cut-in}$ = 3.5 m/s, $\:{v}_{r}$ = 12 m/s and $\:{v}_{cut-out}$ = 25 m/s. Figure 7 shows the power curve for wind turbine where, the classical model doesn’t incpoprate the effect of the ambient temperature on the output power of the turbine. Weibull PDF parameters which are obtained in section *IV* with *k* = 2.995 and *c* = 10.6917 are considered in the results.

Using Eqs. (45–47), The wind power probability distribution for a 2000 kW wind turbine is indicated in Fig. 6. The output wind power was discrete at $\:{P}_{wind}=0$ and $\:{P}_{wind}=\:{P}_{r}$, and continuous probability function between zero and $\:{P}_{r}$.

**Fig. 8:**
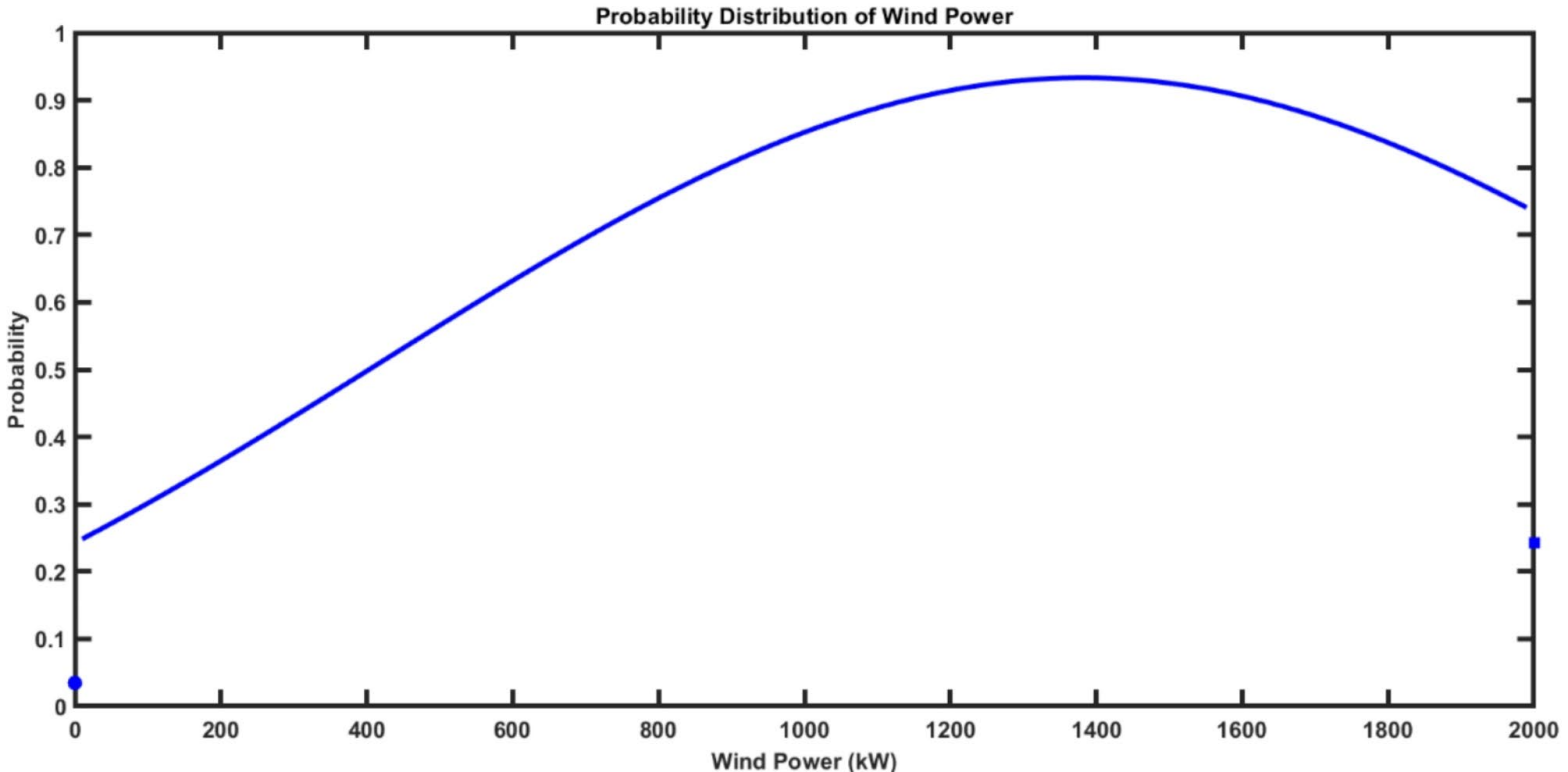
Probability Distribution of Wind Power.

It can be found that the classical model considers only the impact of the stochastic behavior of wind energy that varies with the change of wind speeds. Figure 8 shows the wind power probability distribution function (PDF). However, In practice, the wind turbine output power is affected by the ambient temperature. This effect is presented in the next section.

### Novel wind speed modeling results

**Fig. 9:**
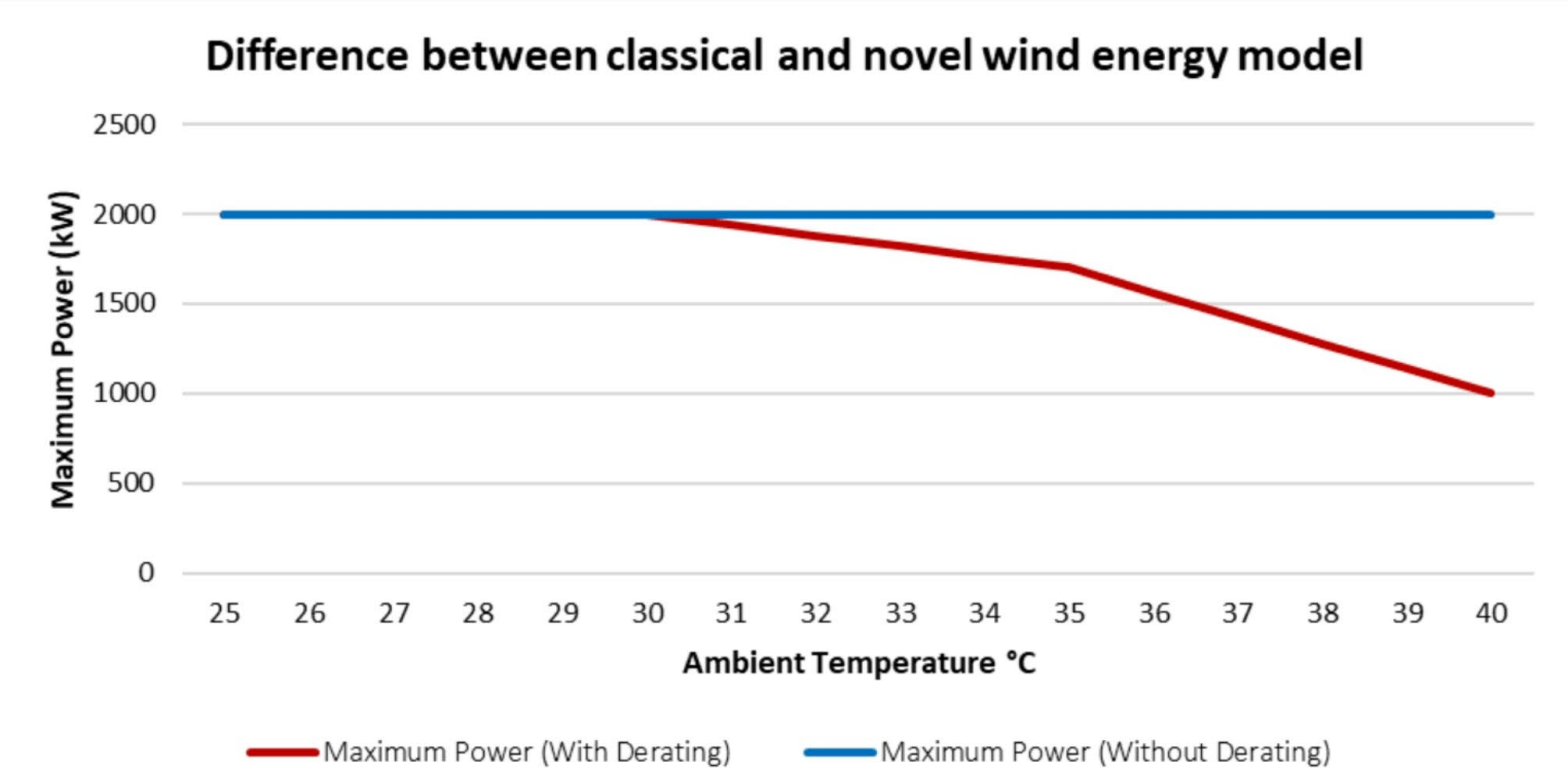
Difference between Classical and Novel Wind Energy Models.

In this part, the impact of the ambient temperature on the wind turbine output power is presented. As discussed earlier, With the increase of ambient temperature the output power of the wind turbine decreases. Figure 9. shows the difference between what was assumed in the classical model against the proposed novel model from ambient temperature prespective. The classical model assumes that the maximum power that can be extracted from a wind turbine is its rated power, the blue line, regardless the ambient temperature value. While the proposed model takes the impact of the ambient temperature in account on the wind turbine output and the wind energy power probability distribution.

Table 3 shows the maximum output power ,$\:\:{P}_{max},$ for G80 wind turbine.

**Table 3:** Maximum output power vs. ambient temperature.

$\:{{T}}_{{a}{m}{b}}\:$°C	$\:{{P}}_{{max}}$ (kW)
30 °C	2000
31 °C	1940
32 °C	1880
33 °C	1820
34 °C	1760
35 °C	1700
36 °C	1560
37 °C	1420
38 °C	1280
39 °C	1140
40 °C	1000

It can be found from Table 3 that the output power decreases significantly with the rise of ambient temperature. For the studied turbine, at 35 °C and 40 °C the output is reduced by 15% and 50% respectively. This significant reduction was not considerd in the classical model.

**Fig. 10:**
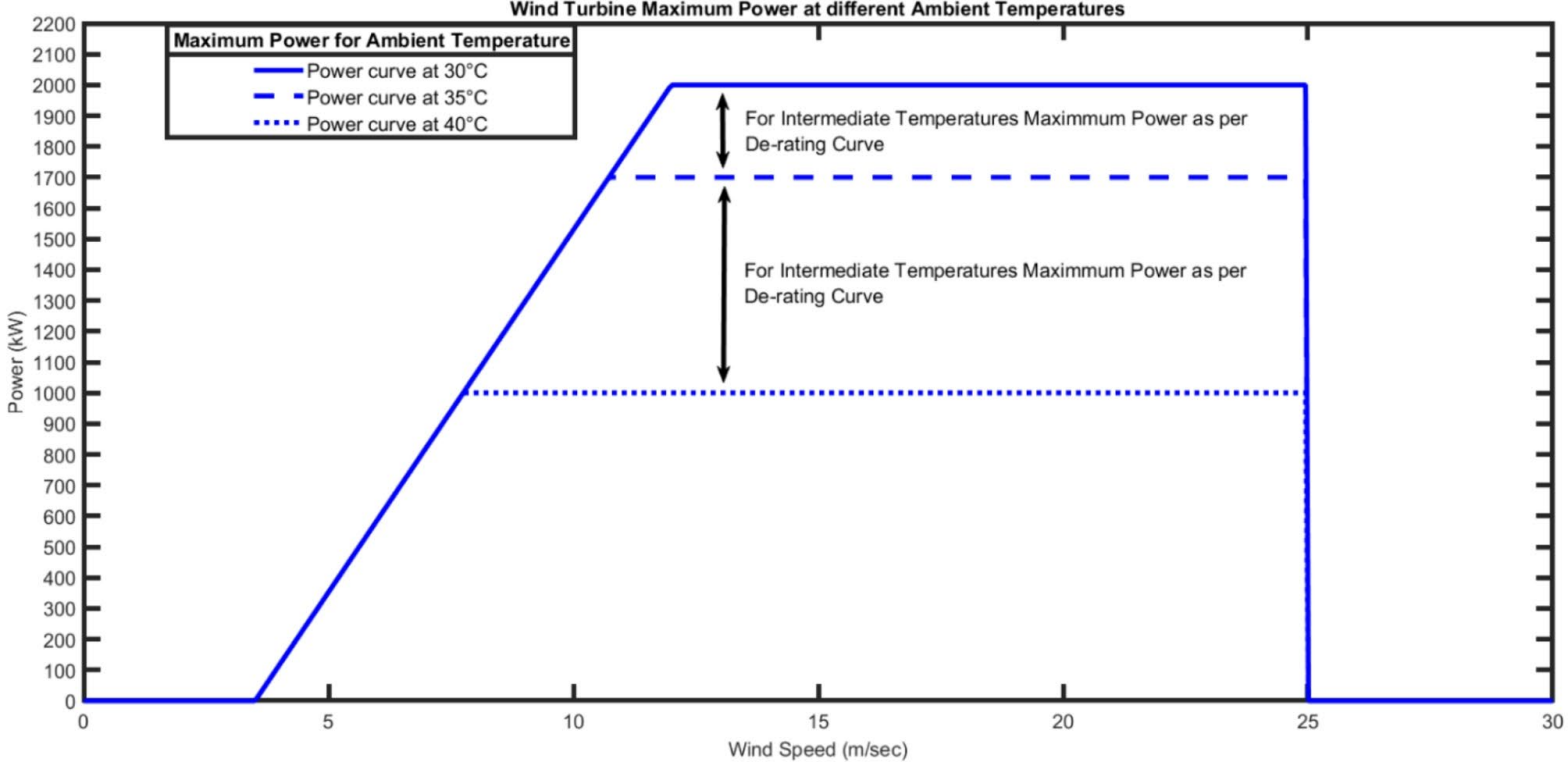
Wind Turbine Power Curve at various Ambient Temperatures.

To validate the proposed model, G80 wind turbine is used with $\:{v}_{cut-in}$ = 3.5 m/s, $\:{v}_{r}$ = 12 m/s, $\:{v}_{cut-out}$ = 25 m/s and $\:\:{P}_{r}$ is 2000 kW. Figure 10 shows the maximum power curve at different ambient temperatures. The wind turbine maximum output power will lie in the reigon depends on the ambient temperature.

**Fig. 11:**
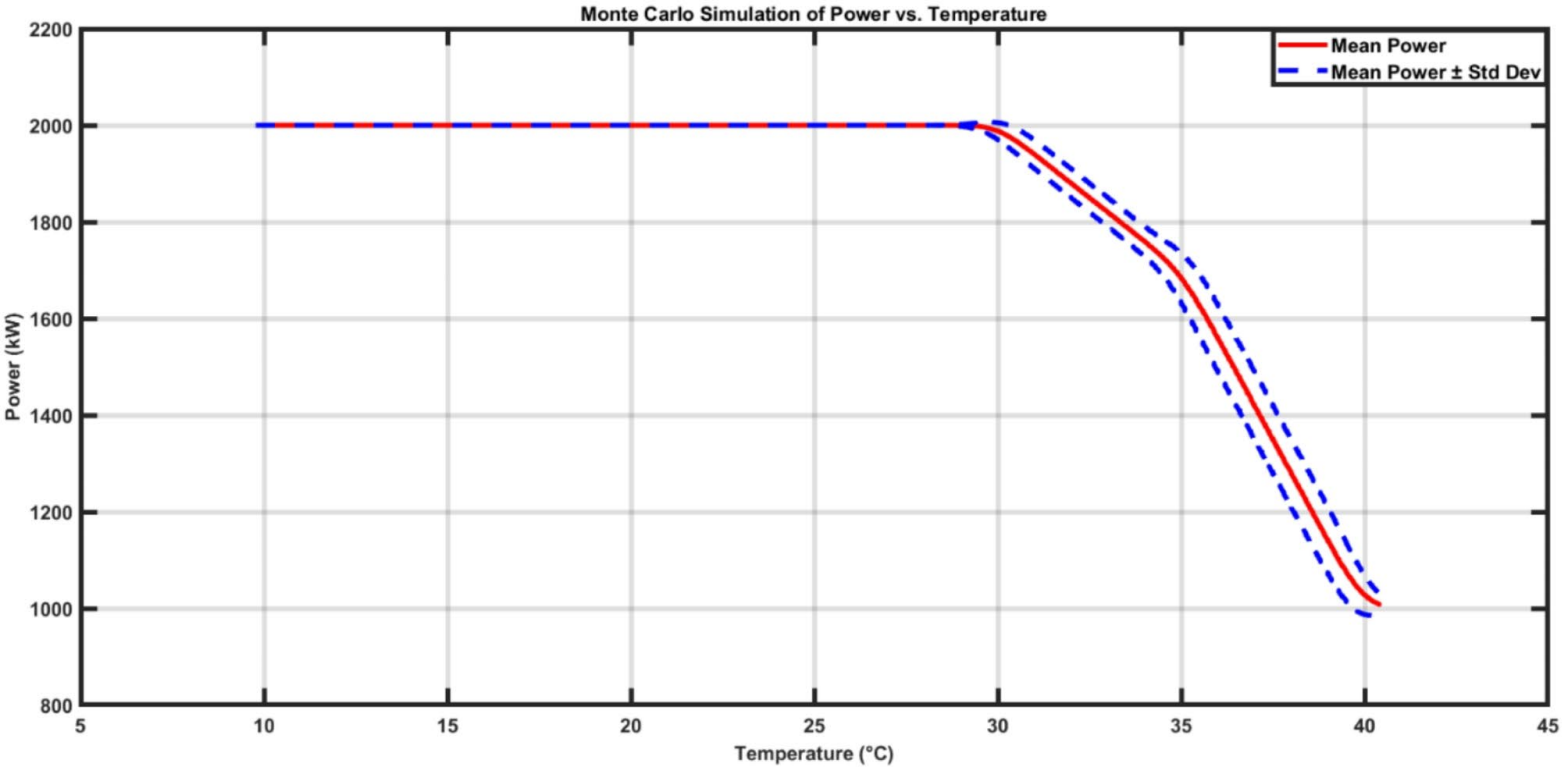
MC Simulation for Power De-rating Curve.

Monte Carlo simulation with sample size *N* = 8000 is used to test the model proposed in this paper where, the sample data are based on the real data from Zafaarana region. Figure 11 shows the de-rating curve which matches to the G80 turbine manufacturer curve. Where, the proposed model provides a correlation of coefficient of 0.998 against the manufacturer De-Rating Curve due to Ambient Temperature.

In order to see the results considering the impact of ambient temperature, G80 wind turbine Power curve and de-rating curve from Figs. (9, 10) are used. Weibull PDF with *k* = 2.995 and *c* = 10.6917 parameters is considered.

**Fig. 12:**
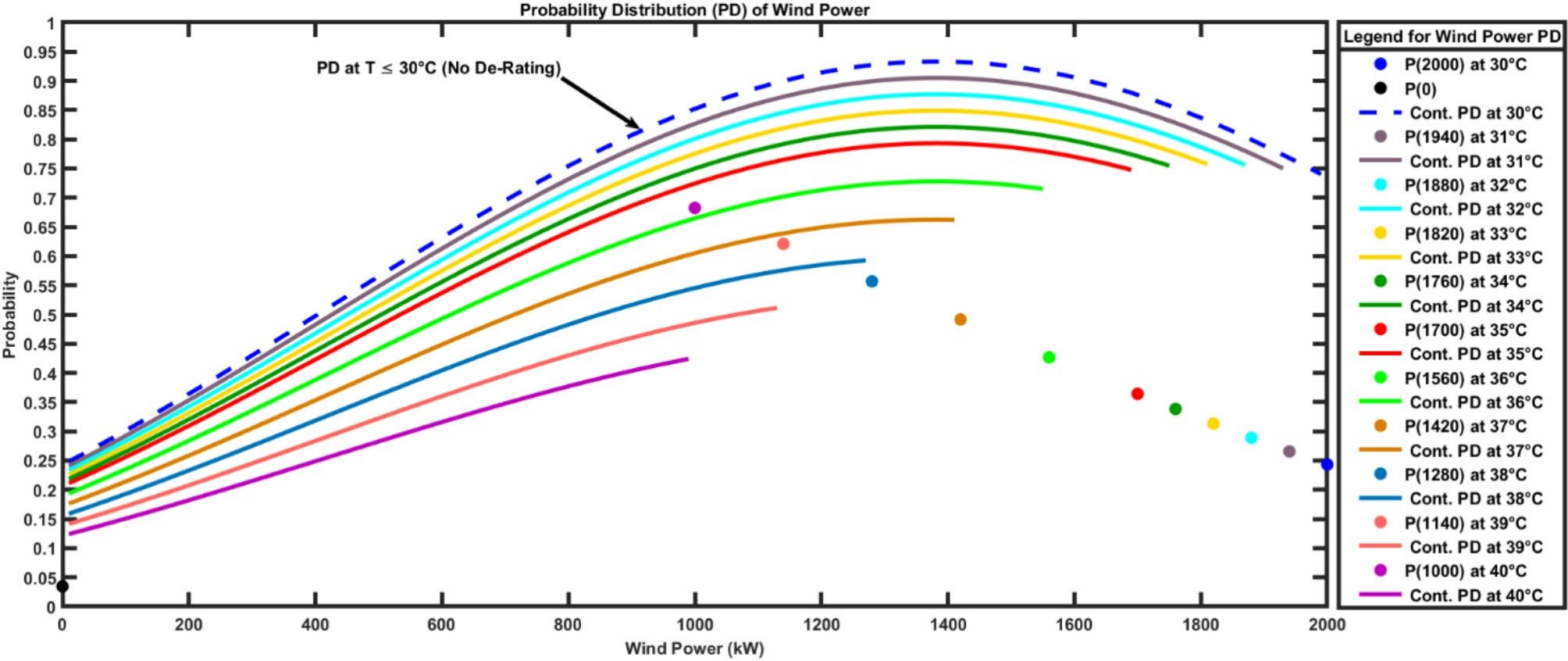
Wind Power Probability Distribution at various Ambient Temperatures.

By using Eqs. (51–52), The wind power probability distribution for 2000 kW wind turbine for the ambient temperature range from 30 °C to 40 °C is calculated. Figure 12 shows that the output wind power was discrete at $\:{P}_{wind}=0$ and $\:{P}_{wind}={P}_{max}$. While it’s continuous between 0 and $\:{P}_{max}$. It can be observed that power probability distribution varies depending on the ambient temperature which would have an impact on the assessment of wind energy in power systems.

Table 4 shows the variations in the probability of maximum output power considering the ambient temperature effect.

**Table 4:** Probability of maximum output power at different ambient temperatures.

$\:{{T}}_{{a}{m}{b}}$ (°C)	$\:{{P}}_{{max}}$ (kW)	$\:{{v}}_{{max}}\:$ (m/s)	$\:{{f}}_{{w}}\left({P}\right)\left\{\:{{P}}_{{w}{i}{n}{d}}={{P}}_{{m}{a}{x}}\right\}$
30 °C	2000	12	0.2432
31 °C	1940	11.745	0.2656
32 °C	1880	11.49	0.289
33 °C	1820	11.235	0.3133
34 °C	1760	10.98	0.3384
35 °C	1700	10.725	0.3643
36 °C	1560	10.13	0.427
37 °C	1420	9.535	0.4916
38 °C	1280	8.94	0.557
39 °C	1140	8.345	0.621
40 °C	1000	7.75	0.6828

It can be deduced from Table 4 that with the increase of the ambient temperature temperature, the probability of having reduced output power increases. At 35 °C, it’s almost 36% probability to have a maximum power of 1700 kW, which is 85% of the wind turbine rated power, even if the wind speed excceds its wind turbine rated speed i.e. 12 m/s. At 40 °C, it’s almost 68% probability to have a maximum power of 1000 kW which represents 50% of the wind turbine rated power even if the wind speed exceeds the wind turbine rated speed to respect the capabilities defined by the turbine manufacturer. Accordingly, the proposed model reflects the practical capabilities for wind turbines. The application of the proposed novel model can help system operators assess the overall impact of integrating wind energy on power flow, fuel cost, voltage stability, and carbon emissions in electrical networks. From system operator side, the power flow shall be assessed at high temperature to adjust the generation from conventional power plants and reserve scheduling to minimize the operating costs during high temperature seasons. From wind farm operator, maximum expected generated power during high temperature seasons shall be prepared such that the set points for system operators be more accurate.

## Conclusion

This paper focuses on the effects of climate change and the increase in ambient temperature on the efficiency of wind energy. The contribution of this work is presented as a novel wind energy model that includes the effect of the ambient temperature on the performance of the output power of the wind turbines where, the proposed model achieves an accurate representation for the behavior of wind turbines considering the impact of ambient temperature. To prepare the model, three AI methods, AO, EDO and EO were used to parameters for various PDFs. EDO and EO managed to achieve better results for the objective function defined. The result showed that Weibull PDF provides 3% and 6.8% higher Coefficient of Correlation as well as 56% and 64% lower RMSE compared to Gamma and Lognormal PDFs. Moreover, Mixed-three Weibull PDF provided 1.5% and 0.5% higher Coefficient of Correlation as well as 55% and 42% lower RMSE compared to single and mixed two PDFs respectively for Zaafarana region. In classical wind energy modeling, the impact of ambient temperature was not considered, the model only focusd on the stochastic behavior of the wind speed only which is a major drawback. To improve the assessment of wind energy in electrical power systems, this paper proposed a novel model incorporating the impact of ambient temperature. Depending on each manufacturer, each wind turbine has its de-rating curve due to ambient temperature. In this paper, G80 2 MW wind turbine de-rating curve was used in the validation of the proposed model. The principle equations and de-rating curve showed that the maximum output power from a turbine can be reduced significantly due to high ambient temperature independently from wind speed. Where, the output power could reach 50% of its rated value at high ambient temperature. The results for the classical model showed that the probability of having rated power is 24.3% when the wind speed is equals or higher than the rated wind speed. For the proposed model, The results showed that the wind turbine output power changes tremendously with the rise of ambient temperature. Where, it can be observed that 85% of the wind turbine rated power can be extracted when the ambient temperature reaches 35 °C, while it reaches 50% when the ambient temperature reaches 40 °C. The probability of having 50% of the turbine rated power is 68% even if the wind speed reaches the wind turbine rated speed. The output power remains de-rated to respect the limitations defined by the turbine manufacturer. Moreover, Monte Carlo Simulation was used to test the proposed model against the manufacturer curve. The results showed that the proposed model achieves a coffieicent of correlation of 0.998 against the manufacturer’s de-rating curve. Thus, the proposed model provides better accuracy and closer performance to the wind energy conversion system including the limitations in wind tubrines compared to the classical models which is contribution of this research. It is worth mentioning that the application of the proposed novel model can be extended to help system operators assess the overall impact of integrating wind energy on power flow, fuel cost, voltage stability, and carbon emissions in electrical networks.

## Data Availability

The datasets used and/or analyzed during the current study are available from the corresponding author on reasonable request.
